# Guide plate technology improves traditional fluoroscopy on the fixation of AO/OTA 33-C distal femur fractures with integrated nail–plate double distalis entry technique: a retrospective comparative study

**DOI:** 10.3389/fmed.2026.1976071

**Published:** 2026-10-28

**Authors:** Yongde Wu, Bo Chen

**Affiliations:** Department of Orthopedics, Yichang Central People’s Hospital, The People’s Hospital of China Three Gorges University, Yi Chang, Hubei, China

**Keywords:** distal femoral fractures1, dual-plate fixation3, knee fractures4, nail plate construct2, surgical complication5

## Abstract

**Introduction:**

NPC (nail–plate construct) has become the surgical mainstay for AO/OTA 33-C-type femoral fracture. Two NPC (nail–plate construct) techniques have been developed: a dedicated surgical guide device-assisted technique (NPC-GD) which adjusts conventional intraoperative fluoroscopy to lead to more accurate reduction of fractures and safer implant placement than ordinary fluoroscopy-guided technique, and a regular fluoroscopy-guided technique (NPC-GF). Aim: Comparison of surgical outcomes, quality of life and functional recovery in patients with AO/OTA 33-C-type femoral fractures using either NPC-GD or NPC-GF.

**Methods:**

A retrospective comparative analysis was conducted among 161 cases enrolled from Jan. 2020 to Jan. 2025 including 86 cases in NPC-GD group and 75 cases in NPC-GF group. Operative time, operation blood loss, fluoroscopy frequency, rate of malunion, healing time, weight-bearing time, knee range of motion and HSS knee score were statistically compared. Outcome evaluations were all performed in a blinded fashion.

**Results:**

This final analysis included 161 patients: 86 in the NPC-GD group and 75 in the NPC-GF group. NPC-GD group had a shorter operative time (*P* = 0.006), intraoperative (*P* = 0.004) and postoperative (*P* = 0.003) blood loss, fewer numbers of intraoperative fluoroscopic images (*P* = 0.034), shorter time to radiographic fracture union (*P* = 0.037), earlier onset of walking at final follow-up (*P* = 0.001), earlier achievement of full weight-bearing at final follow-up (*P* = 0.009), greater final knee range of motion (*P* = 0.009), better HSS knee scores (*P* = 0.002).

**Conclusions:**

NPC-GD is the optimized fluoroscopy technique; it reduces the surgical damage and excess irradiation. NPC-GD can facilitate fracture healing and achieve satisfactory repair of knee function, which is a preferred method of AO/OTA 33-C distal femoral fracture internal fixation.

## 1 Introduction

Recent studies report the overall incidence of distal femur knee fracture as 8.7 per 100 000 person-years. Comminuted distal femoral fracture have challenging treatment owing to disagreement of effective fixation technique. Rising with high energy motor vehicle collisions and elderly osteoporotic fractures of distal femur in native and prosthetic knees keep increasing (1). AO/OTA classification type 33-C (formerly 3C) fractures are complex articular fractures that involve both the medial and lateral condyles and are combined with disruption of the medial and lateral cortices. These fractures are usually associated with bicondylar comminution, medial femoral condyle defects, osteoporosis and large segments of distal femoral fracture fragmentation. Because they play critical roles in weight-bearing and knee function, operative stabilization with internal fixation is a reasonable standard of care (2). Conventional fixation techniques for complex distal femoral fractures include locked plating, retrograde intramedullary nailing, rarely followed by hybrid or external fixations (3, 4).

Yet, these approaches have relatively high complication rates: nonunion rates are up to 19% of cases (5), failure of implants at approximately 20% (6). There are relatively high rates of infection following dual plating (7) and one-year mortality in the elderly is 21% (8).

The modified dual-construct fixation technique has gradually gained clinical and research interest (9). Specifically, either adjunctive medial plating (‘double plating’) or the nail–plate construct (NPC) has great advantages in accelerating the time to weight-bearing and in reducing the risk of implant failure and nonunion (4, 9, 10). However, double plating is inherently eccentric fixation (11), and in certain types of fractures, including those of limited comminution, double plating rarely yields proper nail–bone purchase and remains vulnerable to varus and valgus loading (12). More importantly, medial insertion of a plate requires extensive soft-tissue dissection, increasing the operative invasiveness and potentially compromising periosteal and metaphyseal blood supply (7, 13).

Although the nail–plate construct (NPC) has shown favourable short-term results regarding restoration of alignment and early loading potential of the implant (14, 15), implant interference between the intramedullary nail and plate screws (16) as well as partial and often eccentric cortical interference of the locking screws, compromise the conduct of surgery and stability of the construct as a whole.

Georgios and collaborators introduced the fluoroscopy-guided nail–plate construct (NPC-GF) (9): nail–plate construct achieved by locking compression condylar screw connecting nail and plate, and uses the ‘perfect circle’ nail–plate interlocking technique conducted by fluoroscopy positioning in real time, accurate distal interlocking is achieved; single-cortical proximal screw fixation was used in lateral plate. Li and collaborators (17) then used the guide device–assisted nail–plate construct (NPC-GD) to replace intraoperative fluoroscopy for the distal hitting target, and the purpose of our study was to report the AO/OTA 33-C Distal Femur Fractures fixed by the NPC-GD or the NPC-GF outcome; and another aim of this study is to analyse the association between the two fixed methods and outcome functional score.

### 1.1 Study design

A retrospective cohort study was performed in 161 patients with AO/OTA 33-C-type distal femoral fractures treated in our centre between January 2020 and January 2025. All patients were consecutively enrolled and treated with internal fixation for AO/OTA 33-C-type distal femoral fractures with either internal fixation technique of the NPC-GD or the NPC-GF according to the surgeon's preference. There was no impact from equipment shortages, and patients were not selectively assigned to either group on the basis. All surgical approaches were performed uniformly by two senior chief physicians and one attending physician. The cohort consists of 99 men and 62 women and with a mean age of 57.6 years (range, 28 to 80 years). High-energy injuries such as collisions with motor vehicles and falls from heights were responsible for most of the fractures. Of 161 patients, 86 received guide device–assisted nail–plate fixation (NPC-GD, Figure 1). and 75 received fluoroscopy-governed nail–plate fixation (NPC-GF, Figure 2). The study was reviewed and approved by the Institutional Ethics Committee (Approval No.: E20244801) and all patients gave written informed consent.

**Figure 1:**
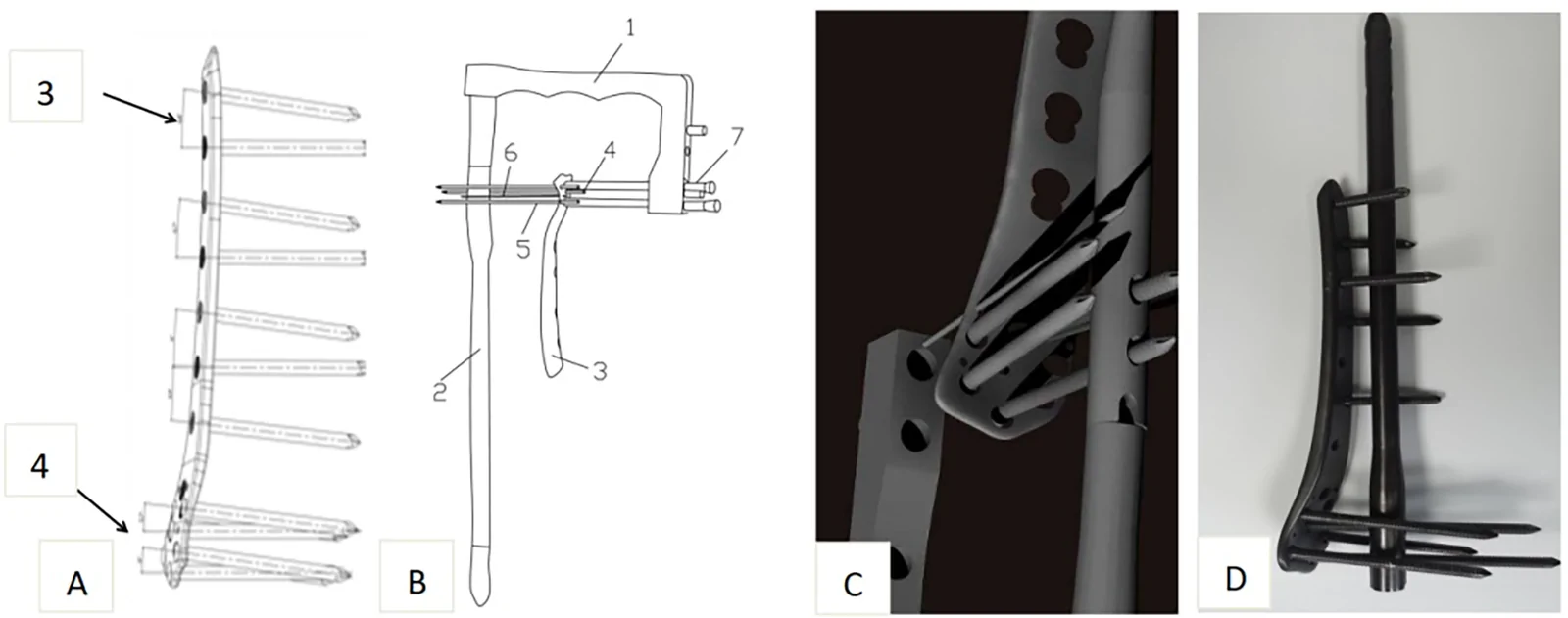
Observation group: NPC-GD. **(A)** Specific lateral locking plate: 3: Proximal eccentric locking screw hole, 4: Two plate-screw interlocking screw holes at the condyle. **(B)** Schematic diagram of the plate-screw interlocking system under guide plate technology: 1. External guide, 2. Intramedullary nail, 3: Proximal eccentric locking screw hole, 4: Two plate-screw interlocking screw holes at the condyle, 5. Reduction-assisted fixation channel (2.0 Kirschner wire can be placed), 6. Locking sleeve, 7. Guide plate locking hole. **(C)** Distribution of distal screws in the plate-screw interlocking system under guide plate technology. D. Schematic diagram of the plate-screw interlocking system after plate-screw interlocking under guide plate technology.

**Figure 2:**
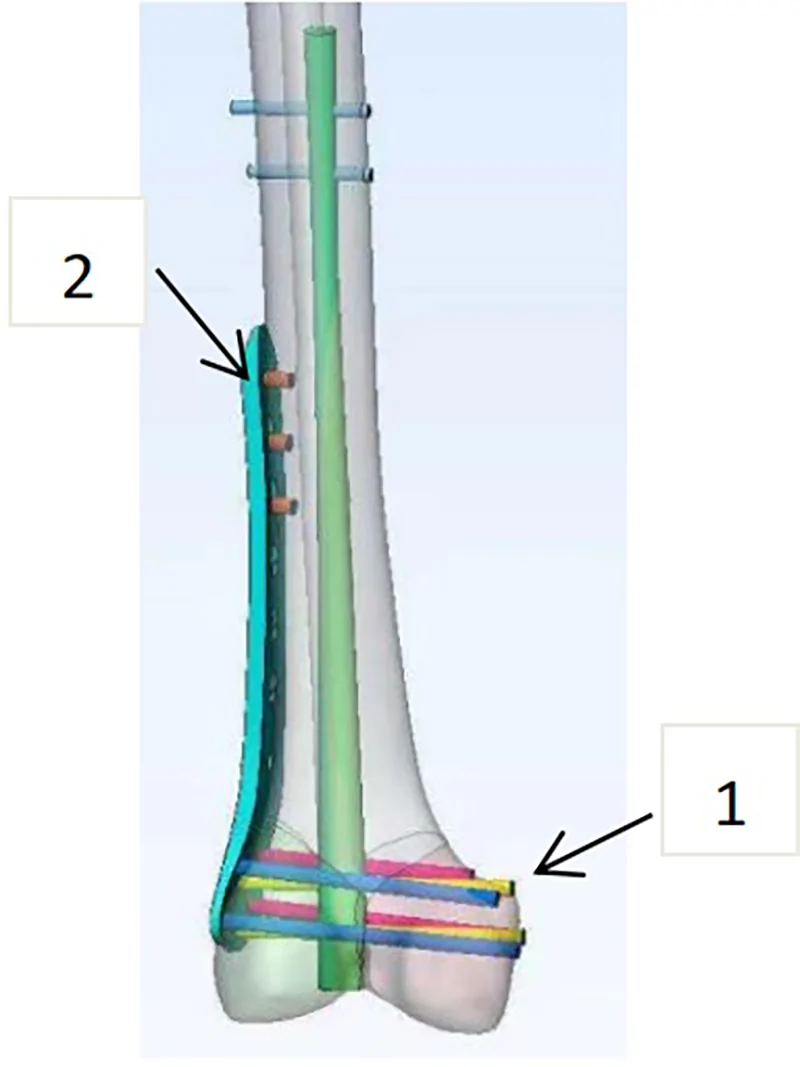
The control group, NPC-GF. Fluoroscopic imaging guidance was employed to achieve precise interlocking fixation in the distal femur (1. yellow screw), while the proximal femur was stabilized using a half-cortical screw (2. orange screw).

### 1.2 Inclusion and exclusion criteria

Criteria for inclusion were: (i) AO/OTA 33-C type of distal femoral fracture and (ii) medical fitness for surgery and ability to follow standard postoperative postrehabilitation regime. Criteria for exclusion were:(i) active local infection at the fracture site, deep vein thrombosis of the lower extremities, major ligament injury, concomitant severe vascular or nerve injury, or bone defects requiring bone grafting; and (ii) incomplete clinical or radiological follow-up data, or a follow-up duration of less than 12 months.

### 1.3 Data collection

Perioperative and functional outcomes (operative time, total intra-operative and postoperative blood loss, volume of allogeneic blood loss, number of intra-operative fluoroscopy images, time to radiographic fracture union, knee range of motion (flexion and extension), complication rate, and Hospital for Special Surgery (HSS) knee score) were prospectively collected and compared between groups. All functional outcomes, were independently evaluated by two specialized orthopedic researchers who were blinded to the surgical grouping information. All postoperative complications including implant-related failure (screw cutout, plate breakage, nail migration), surgical site infection, persistent pain that warranted treatment, etc., were recorded. At final follow-up, a standardized assessment of radiographs (6) was done to assess the reduction quality: excellent (anatomical reduction); good (lateral displacement <3 mm, no shortening, angulation, or rotational deformity); fair (lateral displacement ≥3 mm, shortening <1 cm, angulation/rotational deformity <10°); or poor (lateral displacement >5 mm, shortening ≥1 cm, angulation/rotational deformity ≥10°).

### 1.4 Surgical technique

#### 1.4.1 Preoperative preparations

(i) Confirm absence of active infection and comprehensively treat related soft-tissue injuries, including skin abrasions, bullae, and tissues of necrosis, to maximize conditions for aseptic surgical treatment.(ii) Implement routine preoperative skeletal traction across the tibial tubercle to prevent soft-tissue contracture and facilitate fracture realignment intraoperatively. Meanwhile, the enrolled patients underwent routine color Doppler ultrasound examination of the lower extremity veins to screen for thrombosis in the deep veins of both lower extremities. Those without contraindications were routinely given low-molecular-weight heparin calcium anticoagulant therapy.(iii) Achieve thorough preoperative skin scrub of the entire leg to remove microbial infestations and optimize surgical site sterility.

#### 1.4.2 Surgical procedure

##### 1.4.2.1 NPC-GD group

The procedure was done under either epidural or general anesthesia. Preoperatively, the traction pins were removed. The patient's anterior iliac region and entire lower limb were preoperatively disinfected and draped in a standard manner. A sterile tourniquet was used during the operation. A lateral parapatellar or lateral midline incision was made and the vastus lateralis was split longitudinally to expose the lateral femoral condyle. The patella was medially subluxed or everted to provide direct visibility of the articular surface. For bicondylar or intercondylar fractures, provisional Kirschner wire fixation was carried out on the medial and lateral condyles: avoiding interposition with the planned position of the nail or lateral plate. Anatomical reduction of the articular surface and lateral femoral wall was achieved and intra-articular fracture converted to extra-articular metaphyseal fracture fixable by stable plate–nail fixation.

Once a precontoured lateral locking plate had been applied to the lateral condyle and provisionally fixed with 1–2 bicortical short screws, distal fixation was secured, using either full-length screws engaging the medullary canal or, in cases of comminution and/or osteoporosis, short screws (<1.6 cm) with additional auxiliary Kirschner wires. Provisional reduction and limb alignment were confirmed with intraoperative fluoroscopy. The incision was further extended distally as needed, and the patella was fully everted so as to allow direct, visible penetration of the joints, unobstructed, for determination of the definitive articular reduction. The path of the retrograde nail entry was evaluated at the point at which the femoral anatomical axis crossed the joint line. Using fluoroscopic guidance in real time, a guidewire was retrograded into the medullary canal and was aligned exactly with the femoral axis. Reaming then began with reamer size 9 mm and increased sequentially by5-mm increments, until the canal size was greater than the nail selected by 1–1.5 mm. Minor fracture angulation or displacement by reaming was reduced using a condylar reduction clamp. Any interference caused by Kirschner wires was either detached or replaced to allow the nail trajectory. Finally, the retrograde intramedullary nail was then inserted over the guidewire, advanced over the guidewire by 40 to 60 mm beyond the distal part of the plate and fine-featured in depth and rotation with a nail-targeting sleeve (Figure 1B.7). Distal locking was done by insertion of 2 fully threaded screws through holes for interlocking in the plate (Figure 1A/B.4) and these are allowed to engage freely and bicortically. The eccentric locking design of the proximal screw holes enabled the proximal locking screws to avoid the intramedullary screws.After the two distal interlocking screws had been secured, proximal locking was completed using interlocking screws placed through eccentric holes in the plate, allowing bicortical screw without obstructing the intramedullary passage. Proximal nail locking was done by using the on-board targeting. Constructs and the axial force line of lower extremity anatomy were checked fluoroscopically.

Any temporary fixation device (Kirschner wires, short cortical screws) was removed, and long screws were inserted as required for supplementary fixation. Final surgical fluoroscopy assessment ensured excellent implant placement, satisfactory fracture reduction and biological-mechanical locking.

##### 1.4.2.2 NPC-GF group

Surgery was performed under epidural or general anesthesia. After removal of the traction pin, standard aseptic preparation and draping of anterior iliac crest and lower limb were done. A sterile tourniquet was used during the operation. Extended lateral anterior approach was used to expose the distal end of femur. After direct view and anatomical reduction of articular and metaphyseal component, provision pin fixation was done first. IM reaming was done before insertion of nail. Standard lateral locking plate was applied after reduction, provisionally nailed. Real-time lateral fluoroscopy was done to adjust the positions of plate and nail in iterations to obtain perfect alignment and bone–imprint best fit.

‘Perfect circle’ technique was used to rotate the distal locking holes of the nail in line with the distal interlocking holes of the plate, ensuring that at least one distal locking screw was passed coaxially through both the nail and the plate (a rigid integration of two constructs). Proximal fixation of the plate was done with partially threaded cortical or locking screws. Locking of the proximal nail was done using a targeting device. Final fluoroscopy images were used to confirm the implant position, the interlocking precision, the fracture reduction.

In both groups, we used fluoroscopy to monitor the recovery of the lower extremity force line during the operation. For cases without obvious anatomical landmarks, after maintaining the force line reduction with temporary fixation, fluoroscopy was used to confirm the mechanical axis of the lower extremity to avoid varus/valgus deformities. For type 33-C comminuted fractures of the distal femur, in addition to the standard anteroposterior and lateral fluoroscopy of the knee joint, full-length lower extremity fluoroscopy is added to evaluate the mechanical axis of the femur and the tibiofemoral Angle. All fluoroscopic images are interpreted in real time by the surgeon for intraoperative resetting and force line adjustment.

#### 1.4.3 Postoperative management

Postoperative care: Administer routine prophylactic antibiotics during perioperative period, continued to 24 h after surgery. During the hospital stay, low-molecular-weight heparin calcium was administered and rivaroxaban was taken orally after discharge to prevent thrombosis until 5 weeks after the operation. Postoperative radiographs (x-ray and CT scan) are taken on the 3rd day after surgery. Drainage tube was removed usually within 48 h after surgery. Passive progressive knee joint mobilization with continuous passive motion (CPM) machines and active rehabilitation exercises start from the 3rd postoperative day. Patients can do walking with crutches and non-weight-bearing (NWB) on the operated leg. Partial weight-bearing was started when radiographically blurring line of fracture appears. Full weight-bearing was allowed only after correct, continuous callus production appears on follow-up x-ray radiographs, generally 2 to 3 months after surgery. Crutches were gradually dropped as functional rehabilitation was progressing. Regular follow-up included serial x-ray examination and range of motion (ROM) examination at 6 weeks, 3 months, 6 months, 1 year, and 2 years after surgery.

### 1.5 Statistical analysis

Continuous variables are presented as mean ± standard deviation of normally distributed data and median (interquartile range) of non-normally distributed data. Categorical variables are presented as frequency (percent). For comparison of continuous data between groups, between groups with different assumptions were done using an independent-sample t-test when data fit to assumption of normality (based on Shapiro–Wilk test) and homogeneity of variance (based on Levene's test), otherwise Mann–Whitney U test was used; categorical variables were analysed using the chi-square test or Fisher's exact test, as appropriate. A *P* value of two-sided *P* < 0.05 was considered to be significant.

### 1.6 Demographic data

Follow-up periods of two groups were between 18 and 32 months. The median follow-up periods were about 24 months in observation group and about 22 months in control group, and the difference was not statistically significant. The time interval from the accident to the operation from 5 to 7 days, and its mean value was 5.3 days. There was no statistically significant difference in the basic demographic, fracture classification or clinical characteristics between two groups (*P* > 0.05). Baseline demographic data of both groups were summarized in Table 1.

**Table 1:** Demographic data of included patients.

Variables	Observation group (*n* = 86)	Control group (*n* = 75)	*P* value^a^
Age (years), mean ± SD	72.３±17.２	65.７±2.2	0.576^b^
Female ratio, n	55／31	50／25	0.781^c^
Time-to-surgery（d),mean ± SD	5.6 ± 2.２	6.３±3.3	0.883^b^
Comorbid diseases: Hypertension/coronary heart disease/others	9	7	0.631^c^
BMI (kg/m2), mean ± SD	24.8 ± 3.8	23.2 ± 2.1	0.687^b^
Hospitalization (d), mean ± SD	14.55 ± 2.34	15.11 ± 3.23	0.783^b^
Follow-up (m), mean ± SD	24.51 ± 6.54	22.11 ± 5.23	0.791^b^
AO/OTA 33-C1/C2/C3	21/41/24	18/37/20	0.735^c^/0.831^c^/0.818^c^

There was no significant difference.

a *P* < .05 was considered statistically significant. ^b^t test. ^c^Pearson *χ*^2^test.

### 1.7 The condition during the perioperative period

All surgeries in the 161 patients were finished successfully without any major surgeries, such as vascular and infectious injuries and nerve injuries. All incisions healed up primary good, with no skin hematoma, wound dehis-cence and necrosis of tissues existed. Postoperative radiographs showed satisfactory fracture reconstruction and stable internal fixing without screw affecting entering the joints. The observation group had a good advantage over the control group, such as a shorter surgery time, less intraoperative blood loss, less postoperative bleeding and C-arm fluoroscopic condition days, all with a significant difference (*P* < 0.05), while the surgical results of two groups are shown in Table 2.

**Table 2:** Comparison of surgical data between the two groups.

Variables	Observation group (*n* = 86)	Control group (*n* = 75)	*P* value^a^
Operation time (min, x ± s)	110.89 ± 18.78	166.28 ± 23.73	0.006^b^
Total hospital stay (days, x ± s)	12.89 ± 1.63	12.72 ± 1.70	0.653^b^
Intraoperative blood loss (ml, x ± s)	385.36 ± 80.45	515.36 ± 80.51	0.004^b^
Postoperative drainage blood loss (ml, x ± sl)	99.03 ± 23.70	146.72 ± 15.70	0.003^b^
C-arm fluoroscopy usage frequency D- (times, mean ± SD)	8.17 ± 2.24	16.56 ± 3.20	0.034^b^

There were statistically significant differences in Operation duration, intraoperative blood loss, postoperative drainage blood loss, and the number of fluoroscopy with C-arm.

a *P* < .05 was considered statistically significant. ^b^t test.

### 1.8 Follow-up results

Following surgery, patients in both observation and control groups gradually transitioned from non-weight-bearing to partial weight-bearing and then to full weight-bearing and participated in the same rehabilitation programs. Knee joint function also gradually improved with time and no recurrence of fracture or other activity limitation during the follow-up time period. The time to initiation of walking and achieving full weight-bearing was shorter in observation groups than that in control groups (*P* < 0.05). At last follow-up, the ROM and HSS score in observation group were significantly higher than those in control group (*P* < 0.05), but there was no statistical difference in residual deformity between the two groups (*P* > 0.05).

With respect to postoperative complication, no internal fixation failure or secondary operation occurred in either group after operation. In the observation group, wound-related postoperative complications occurred in 3 patients, and in the control group, 2 patients had these types of complications; all recovered without recurrence after cleaning and debridement, symptomatic management, and there was no difference between the two groups. Follow-up data for the two groups are shown in Table 3.

**Table 3:** Comparison of follow-up data between the two groups.

Variables	Observation group (*n* = 86)	Control group (*n* = 75)	*P* value^a^
Time to weight-bearing（d,*x* *±* *s*）	18.72 ± 2.25	25.84 ± 4.83	0.001^b^
Time to full weight（w,*x* *±* *s*）	7.17 ± 1.98	14.6 ± 3.49	0.009^b^
knee joint range of motion（^o^_,_*x* *±* *s*	114.17 ± 14.57	100.40 ± 18.48	0.009^b^
HSS score(score，*x* *±* *s*)	85.617 ± 3.87	73.36 ± 7.40	0.002^b^
Shortening deformity [case]	6	4	0.999^cc^
varus-valgus deformity[case]	0	0	-
postoperative complications[case]	3	2	0.899^c^

There was a statistically significant difference inTime to weight-bearing, full weight-bearing time, knee joint range of motion, HSS score, shortening deformity and varus-valgus deformity. ^a^*P* < .05 was considered statistically significant.^b^t test. ^c^Pearson *χ*2test.

Three days after operation, x-ray examination found no difference between the groups in the quality of fracture reduction. At the end of follow-up period, radiographic evidence for fracture healing were all proven for the fracture of all the patients. Mean time for fracture healing in the observation group was much shorter than that in the control group (*P* < 0.05) (Table 4). The radiography images of a patient in the observation group are shown in Figure 3, the images in control group in Figure 4.

**Table 4:** Comparison of imaging data between the two groups.

Variables	Observation group (*n* = 86)	Control group (*n* = 75)	*P* value^a^
Fracture reduction [cases]			0.847^b^
excellent	72	68	
fair	11	5	
shortening deformity	3	2	
poor.	0	0	
Time for fracture healing [cases (%)]			0.037^b^
<24week	25	23	
>24week	61	52	

There was a statistically significant difference in healing time of fractures^a^*P* < .05 was considered statistically significant. ^b^Pearson *χ*^2^test.

**Figure 3:**
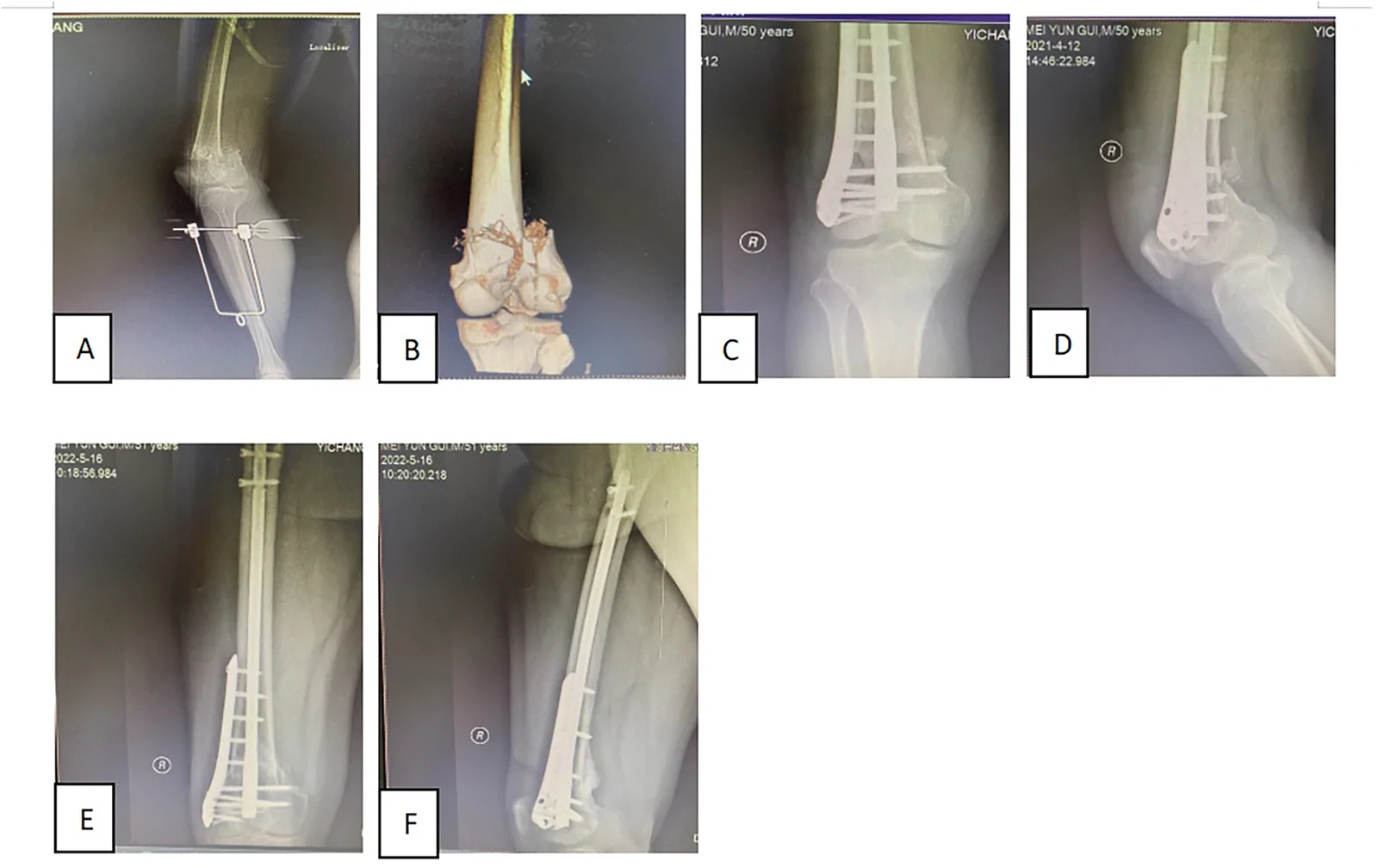
Observation group: reduction of the NPC-GD technology. Representative imaging findings from a 50-year-old male patient with a type 3C distal femoral fracture. **(A)** Preoperative anteroposterior radiograph showing comminuted intra-articular fracture of the distal femur, with temporary skeletal traction applied via the tibial tubercle. **(B)** Preoperative three-dimensional computed tomography (3D CT) reconstruction demonstrating the extent and morphology of the comminuted articular fracture. **(C)** Anteroposterior radiograph obtained three days postoperatively, indicating satisfactory anatomical reduction. **(D)** Lateral radiograph obtained three days postoperatively, confirming adequate alignment and fixation. E. Anteroposterior radiograph at two-year follow-up, showing complete bony union. F. Lateral radiograph at two-year follow-up, confirming fracture healing with maintained reduction.

**Figure 4:**
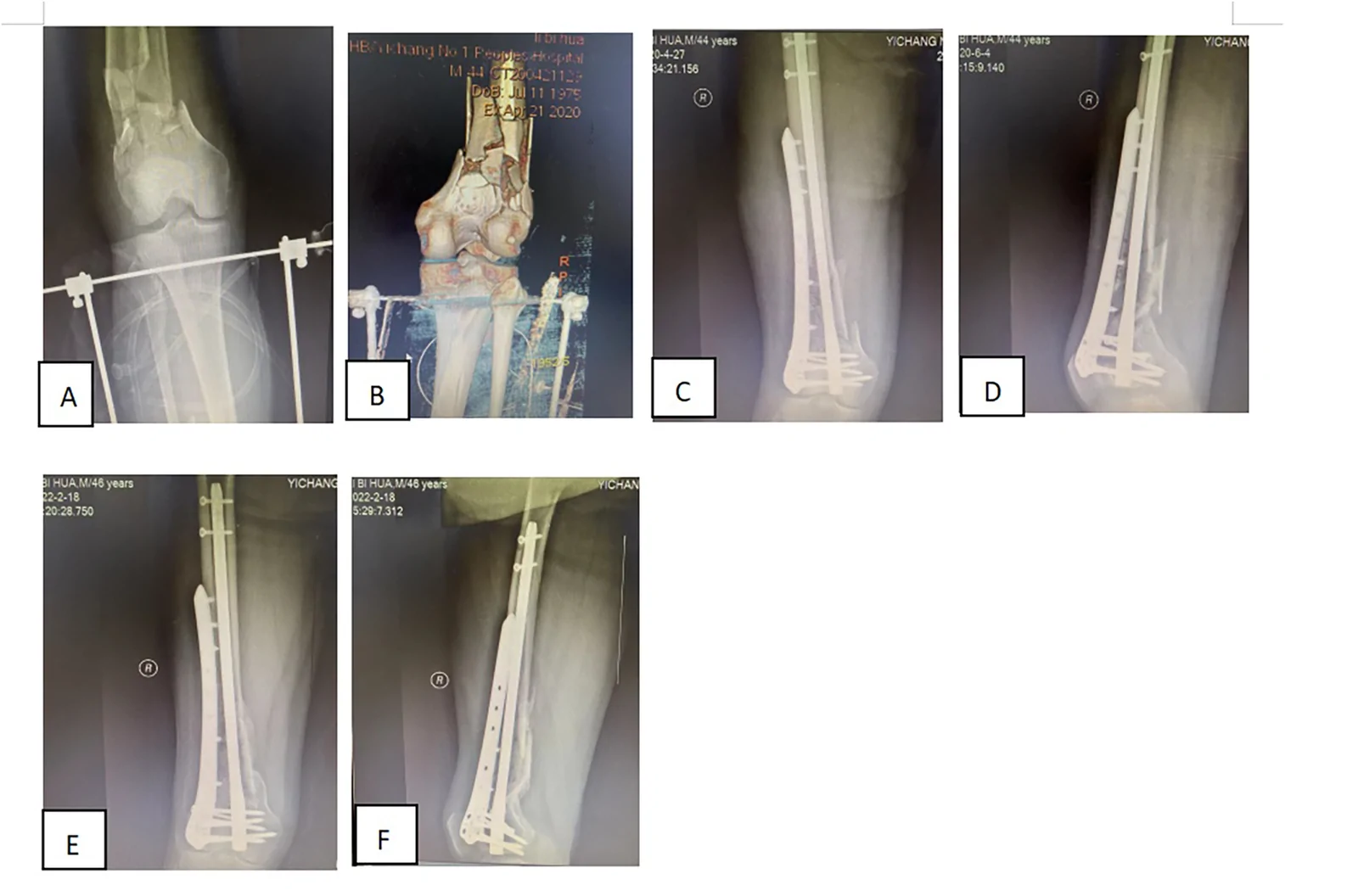
Control group: reduction of the NPC-GF technology. Representative imaging findings from a 44-year-old female patient with a type 3C distal femoral comminuted fracture involving the articular surface are presented. **(A)** Preoperative anteroposterior radiograph demonstrating a comminuted fracture of the distal femur involving the articular surface, with temporary skeletal traction applied at the tibial tubercle. **(B)** Preoperative three-dimensional computed tomography (3D CT) scan confirming the extent of the comminuted intra-articular fracture. **(C)** Postoperative anteroposterior radiograph obtained on postoperative day three, revealing satisfactory fracture reduction and alignment. **(D)** Postoperative lateral radiograph obtained on postoperative day three, confirming adequate reduction. **(E)** Follow-up anteroposterior radiograph taken 15 months postoperatively, demonstrating complete fracture healing. **(F)** Follow-up lateral radiograph taken 15 months postoperatively, confirming consolidated fracture healing.

### 1.9 The key findings

More improved intraoperative outcomes—operative time, intraoperative bleeding, fluoroscopic exposure and time for fracture healing—were achieved in the NPC-GD group compared with the NPC-GF group. Given the complexity of AO/OTA 33-C type distal femoral fractures, approximately 1 in 4 patients treated for distal femur nonunion experienced recalcitrant nonunion despite revision surgery (1, 18, 19). Owing to the anatomically precontoured lateral locking plate used in NPC-GD, which is advantageous for comminuted fractures and osteoporotic bone, was particularly beneficial in converting comminuted 33-C type fractures into more predictable extra-articular metaphyseal (AO/OTA 33-A or 33-B) types, with the ability for the reliable retrograde nail fixation in a subsequent operation; most critically, the dedicated surgical guide allows accurate, fluoroscopy-free, bicortical distal interlocking- nail–screw interference eradication and iterative image-assisted lock adjustments eliminated.

By contrast, in the NPC-GF technique, the intramedullary nail is inserted pre-application of the plate (9, 20). So anatomical reduction and fixation of the plate need to go through long duration of the operation, which results in a large amount of blood loss and soft-tissue injury (21), and also coaxial alignment of plate and intramedullary nail is needed to introduce the ‘‘perfect circle’’ technique for distal lock, thus fluoroscopic examination is needed to be repeated times and time-long surgical operation operation further increase the radiation exposure and infection time of the operation.

Complex AO/OTA 33-C-type distal femoral fractures require anatomical reduction and rigid fixation to enable early mobility. The results of our NPC-GD group showed shorter time to radiographic fracture union earlier commencement of walking at last follow-up, earlier attaining full weight-bearing, larger final knee joint ROM and higher HSS knee scores at final follow-up. The previous biomechanical and clinical studies showed that optimal fracture healing requires controlled interfragmentary micromotion at the fracture site, with an optimal strain magnitude of 2%–10% (4, 22). Inappropriate rigid fixation may lead to stress shielding, reduced mechanical stimulation of fracture site and thus failure of callus formation and osseous healing (5). With the NPC-GD construct, distal interlocking is provided by coordinated engagement of the intramedullary nail, plate interlocking holes and a special surgical guide, avoiding the instability usually associated with short eccentrically positioned distal screws from nail-plate interference.

This set-up allows efficient axial load transmission via the IM nail but enormously increases resistance to horizontal shear forces (4). Crucially, the proximal plate segment has no interlocking structures to reduce the stress concentration around the nail–plate junction—thereby maintaining physiologically valid load transmission throughout the length of the femoral shaft. To assure good proximal fixation of the plate free of dependence on the IM nail, the plate has an offset eccentric locking hole allowing a reliable bicortical screw purchase, without compromising on the integrity or the trajectory of the nail。

Collectively, these biomechanical advantages provide for earlier commencement of functional rehabilitation, faster recovery of knee ROM and better fracture healing. Equally, in line with these principles, Liporace FA et al. (23) found that coupling a plate—screw interlocking system to image free guided instrumentation greatly increases axial and torsional stability in complex periprosthetic and periarticular femoral fractures,and can provide stable, balanced fixation to permit immediate weight bearing and early mobilisation.

The guide plate–assisted plate–screw interlocking system has three main advantages: (i) the laterally placed locking plate permits less medial soft tissue dissection and allows exact and reproducible implant placement using a patient-specific surgical guide plate—which makes surgical execution easy; (ii) it combines the biologically stable effect of intramedullary load sharing and lateral plating together and can overcome the worst type of construct failure which is varus collapse from eccentric loading (24), this enhanced stability allowed earlier mobility of the limb (25, 26, 27); and (iii) proximal fixation of the femur is done with bicortical screw insertion through eccentric locking holes to achieve high mechanical stability without dependence on the intramedullary nail.

### 1.10 Limitations

This study also has limitations. First, it is a single centre, retrospective cohort study with a relatively small sample size, resulting in limited power and generalisability. Second, Although outcome assessments were performed by blinded evaluators，there is also limitations in the non-randomization of treatment group assignment, and biases encountered in assessing functional outcomes at follow-up. Third,although current AO/OTA principles recommend rigid integration of the nail and plate to achieve balanced load distribution of the construct (5, 24, 28) and such integration is more common for revision surgery for nonunion after nail–plate construct fixation (28), these constructs also run the risk of being too stiff and inhibiting healing if not kept balanced. The ideal stiffness for fracture healing is not clear (1). Crucially, the effects of such an interlocking configuration on the secondary fracture healing, including callus formation, strain distribution,traumatic arthritis and adaptive loading, need to be ascertained rigorously with experimental biomechanical tests and prospective clinical studies.

## 2 Conclusion

With the popularization of robot navigation technology, the accuracy of screw implantation in complex distal femoral fractures can be further optimized. Future research can evaluate the potential value of robot-assisted screw placement in reducing fatigue failure of internal fixation. NPC-GD technology still has good practicability in most central and even primary hospitals. Our results suggest that the NPC-GD technique optimizes routine intraoperative fluoroscopy and may be able to overcome the important key points of internal implant interference of internal fixator technology in treatment of complex distal femoral fracture. Compared with the NPC-GF technique, the NPC-GD technique gets better clinical effects for AO/OTA 33-C complex distal femoral fractures with less complex operation, less surgical trauma, faster fracture healing and better postoperative functional recovery. However, this single-centre observational study inevitably has selection bias and evaluation bias. The generalizability of our study results is limited and should be verified by large-sampled, multicentre prospective study in the future.

## Data Availability

The datasets presented in this study can be found in online repositories. The names of the repository/repositories and accession number(s) can be found in the article/Supplementary Material.
